# Ammonium-Acetate Is Sensed by Gustatory and Olfactory Neurons in *Caenorhabditis elegans*

**DOI:** 10.1371/journal.pone.0002467

**Published:** 2008-06-18

**Authors:** Christian Frøkjær-Jensen, Michael Ailion, Shawn R. Lockery

**Affiliations:** Institute of Neuroscience, University of Oregon, Eugene, Oregon, United States of America; Massachusetts General Hospital/Harvard Medical School, United States of America

## Abstract

**Background:**

*Caenorhabditis elegans* chemosensation has been successfully studied using behavioral assays that treat detection of volatile and water soluble chemicals as separate senses, analogous to smell and taste. However, considerable ambiguity has been associated with the attractive properties of the compound ammonium-acetate (NH_4_Ac). NH_4_Ac has been used in behavioral assays both as a chemosensory neutral compound and as an attractant.

**Methodology/Main Findings:**

Here we show that over a range of concentrations NH_4_Ac can be detected both as a water soluble attractant and as an odorant, and that ammonia and acetic acid individually act as olfactory attractants. We use genetic analysis to show that NaCl and NH_4_Ac sensation are mediated by separate pathways and that ammonium sensation depends on the cyclic nucleotide gated ion channel TAX-2/TAX-4, but acetate sensation does not. Furthermore we show that sodium-acetate (NaAc) and ammonium-chloride (NH_4_Cl) are not detected as Na^+^ and Cl^−^ specific stimuli, respectively.

**Conclusions/Significance:**

These findings clarify the behavioral response of *C. elegans* to NH_4_Ac. The results should have an impact on the design and interpretation of chemosensory experiments studying detection and adaptation to soluble compounds in the nematode *Caenorhabditis elegans.*

## Introduction

Animals rely on sensory information to respond appropriately to a variety of beneficial and harmful environmental conditions. One such response is chemotaxis, in which an animal samples a continuously changing chemical environment and generates movement toward an attractant [1]. Despite its simple nervous system, the nematode *Caenorhabditis elegans* is able to chemotax to a large number of different attractants including cations and anions, amino acids, alkaline pH, cyclic nucleotides and many volatile organic odorants [1]–[3]. *C. elegans* chemotaxis offers an appealing system to study how the nervous system processes and integrates sensory information with a limited number of neurons.

Chemical compounds that are attractive to *C. elegans* have been classified in several different kinds of behavioral assays. Ward [3] assayed water soluble chemoattraction in radial gradients of attractant. Attraction to anions or cations alone was tested by pairing the tested ion with a counter-ion (ammonium or acetate) that was not attractive under these conditions. These experiments showed that anions (Cl^−^, Br^−^, I^−^) and cations (Na^+^, Li^+^, K^+^, Mg^2+^) are attractive when peak gradient concentrations are 2–20 mM [3]. Similar results were seen in an alternative assay in which worms choose between two streams of liquid containing different attractants. In this assay, weak attraction to ammonium and acetate ions could also be detected [4]. Later, Bargmann and colleagues studied water soluble and odorant chemotaxis in detail [1], [2]. By ablating ciliated amphid sensory neurons with a laser beam, these studies identified the sensory neurons necessary for detecting attractants. They found that water soluble chemotaxis is mediated primarily by the pair of ASE neurons with a minor contribution from ADF, ASG, ASI and ASK [1]. Chemotaxis to odorants is mediated by two other pairs of neurons: AWC and AWA [2]. Thus, *C. elegans* has senses equivalent to taste and smell.

The distinction between taste and smell in *C. elegans* has a morphological correlate. The amphid sensory sensillum contains twelve pairs of sensory neurons, eight of which are directly exposed to the environment. The exposed neurons mainly sense water soluble chemicals. However, there is at least one exception to this; the exposed ADL neurons are important for the avoidance of the odorant 1-octanol [5], [6]. The four pairs of neurons that are not directly exposed to the environment participate in odorant (AWA, AWB, AWC) and temperature sensation (AFD).

Wicks *et al.* [7], and Jansen *et al.* [8], studied attraction to water soluble chemicals with another behavioral assay, the quadrant assay. In this assay, two diagonally opposed quadrants of a plate are filled with an attractive chemical whereas the two remaining quadrants have no attractant. Under these assay conditions, NH_4_Ac is a poor attractant at low concentration (1 mM) but a potent attractant at high concentration (75 mM) [8]. Thus, the attractive properties of NH_4_Ac depend on concentration and the choice of behavioral assay.

Here we show that NH_4_Ac is detected both as a water soluble attractant and as an odorant, and that ammonia and acetic acid individually act as olfactory attractants. We use genetic analysis to show that NaCl and NH_4_Ac sensation are mediated by separate pathways and that ammonium sensation depends on the cyclic nucleotide gated ion channel TAX-2/TAX-4, but acetate sensation does not. Mutant analysis shows that NH_4_Ac is detected by exposed and non-exposed sensory neurons. Furthermore we show that NaAc and NH_4_Cl do not constitute Na^+^ and Cl^−^ specific stimuli under these experimental conditions. Our results clarify NH_4_Ac chemosensation and its molecular basis.

## Results

### Ammonia and acetic acid are volatile attractants

**Figure 1:**
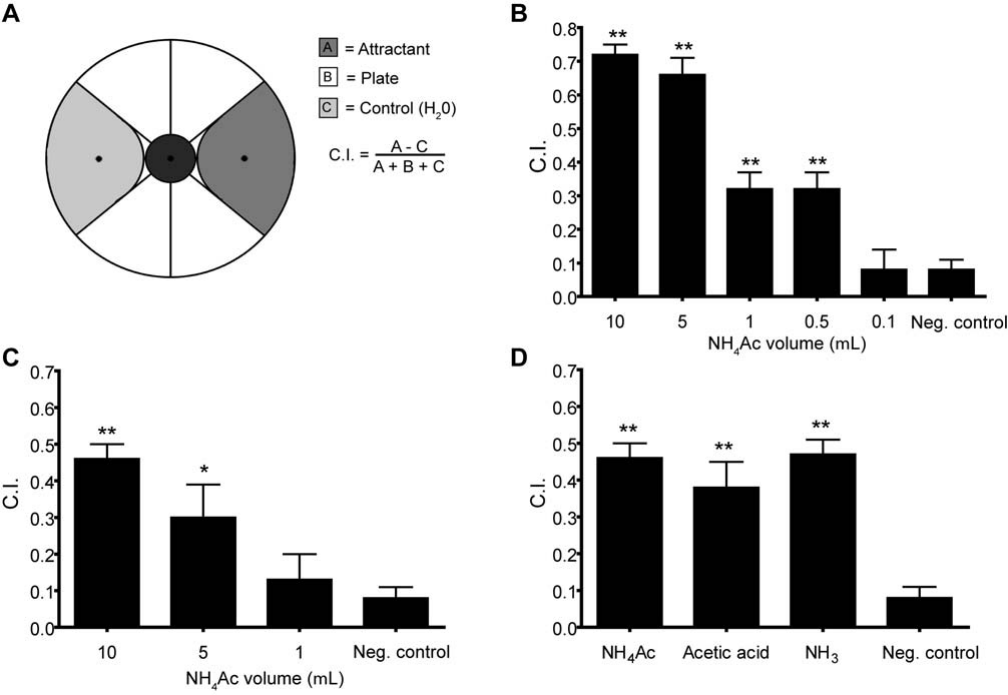
Odorant chemotaxis to NH_4_Ac. (A) Diagram of the assay. Droplets of attractant and negative control solutions were placed on opposite sides of plate at the locations indicated by the eccentric black dots. To manipulate effective NH_4_Ac concentrations, rather than changing NH_4_Ac concentration we changed the droplet volumes as indicated in panels B and C. NH_4_Ac concentration was 7.5 M and adjusted to pH = 6.0 with acetic acid. Sodium-azide was added to immobilize worms reaching either location. Worms were placed at the center of plate and allowed to move for one hour after which worms in zones A, B, and C were counted and the chemotaxis index (C.I.) was computed as shown. (B) Chemotaxis index vs. droplet volume for NH_4_Ac placed directly on the assay plate immediately before the assay. (C) Chemotaxis index vs. droplet volume for NH_4_Ac suspended from the Petri plate lid immediately before the assay. (D) Chemotaxis index for equal volumes (10 µL) of different attractants suspended from the lid. In all panels, H_2_O refers to a negative control in which only water was spotted on the plate. The concentration of NH_4_Ac in the droplets was 7.5 M (pH = 6.0). Each bar represents the mean of at least 8 independent assays. Statistics: * p<0.05 and ** p<0.01 in a one way ANOVA with Dunnet's post test comparing all means to the negative control (H_2_O at both spots).

We previously reported that chemotaxis to the peak of an NH_4_Ac gradient was intact in animals that could not detect NaCl, suggesting that separate pathways exist for detecting these attractants [9]. Because a solution of NH_4_Ac has a characteristic smell, we hypothesized that NH_4_Ac could be detected by an odorant pathway. We assayed odorant chemotaxis (Fig. 1A) by spotting the attractant either on the plate or the lid immediately before the assay. In both conditions wild-type animals accumulated at the attractant source (Fig. 1B, C). Attraction could be toward acetate/acetic acid and ammonium/ammonia or to only one of these compounds. To test this, we assayed attraction to acetic acid and ammonia separately by spotting the attractants on the lid. Both compounds were attractive (Fig. 1D). Thus, *C. elegans* can sense ammonium and acetate as distinct attractants.

### Ammonium-acetate chemotaxis depends on ciliated neurons

To identify signaling pathways that mediate NH_4_Ac sensation we performed chemoattraction assays with well-characterized mutants. We used three types of mutants: (1) cilium structure mutants which have defects in the sensory endings of ciliated sensory neurons, (2) sensory transduction mutants which lack components necessary for signal transduction, and (3) neuron specification mutants which lack transcription factors that are necessary for the correct development and function of specific neurons. Neuron specification mutants can be helpful in identifying candidate cells for functions such as chemosensation. However, experimental results obtained by this approach should be interpreted with caution, because factors such as developmental compensation and residual function of the impaired cells cannot be ruled out.

**Figure 2:**
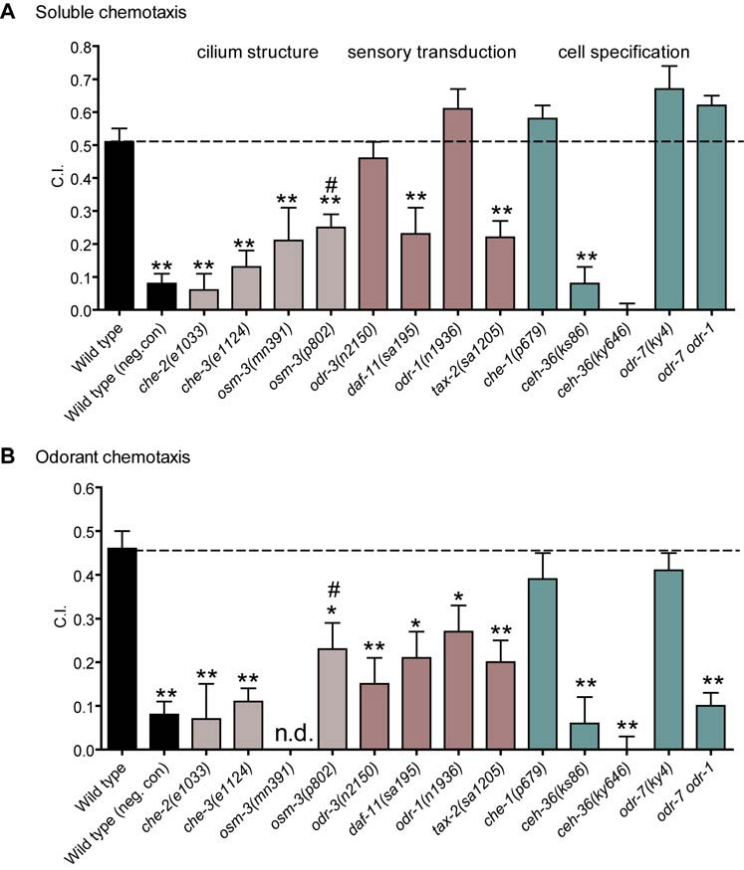
Genetic analysis of chemotaxis to NH_4_Ac presented in water soluble or odorant form. (A) Water soluble chemotaxis assays. Chemotaxis index is plotted vs. strain for assays in which radial gradients of NH_4_Ac were established by diffusion in the agar. (B) Odorant NH_4_Ac assays. Chemotaxis index is plotted vs. strain for assays in which a droplet of NH_4_Ac (10 µL, 7.5 M) was suspended from the lid of the plate. In A and B, each bar represents the mean of at least 8 independent assays; n.d. means no data. Wild type (neg. con) is a negative control assay with no attractant on plate. Statistics: * p<0.05 and ** p<0.01 in a one way ANOVA and Dunnet's post test comparing all means to the wild-type (N2) mean; # p<0.05 in a one-way ANOVA with Dunnet's post test comparing *che-2(e1033)*, *che-3(e1124)*, and *osm-3(p802)* to the negative control.

(1) Cilium structure mutants. Depending on the cells affected, cilium structure mutants display impaired chemotaxis to water soluble attractants (*osm-3*) or to both odorants and water soluble attractants (*che-2* and *che-3*) [2], [7], [10]. *osm-3* is expressed only in sensory neurons with exposed cilia [11] and *osm-3* mutants do not exhibit structural defects in non-exposed cilia [12]. In our assays, two *osm-*3 mutants (*p802* and *mn391)* showed significantly reduced chemotaxis to NH_4_Ac compared to wild-type (Fig. 2), implicating exposed ciliated neurons in chemotaxis to NH_4_Ac. However, *osm-3(p802)* chemotaxed significantly better than the negative control in both water soluble and odorant assays (*osm-3(p802)* vs. negative control, p<0.05). Thus, *osm-3(p802)* chemotaxis was only partially impaired. One way to interpret this is that both exposed and non-exposed ciliated neurons are involved in normal NH_4_Ac chemotaxis. Alternatively, only the exposed neurons are involved, but *osm-3* mutants do not completely eliminate their function. Mutants in *che-2* and *che-3* eliminated chemotaxis to NH_4_Ac (Fig. 2, *che-2*, *che-3* vs. neg. control, p>0.05). These mutants affect both exposed and non-exposed cilia, suggesting that both classes of neurons are involved in sensing NH_4_Ac. However, because these mutants also have more severe structural defects than *osm-3* [7], [10], [12], it does not exclude the possibility that only exposed neurons are involved (but see below).

(2) Sensory transduction mutants. These mutants show a variety of phenotypes, from broad defects in many sensory modalities to defects in the response to a single odorant [13]. The cyclic nucleotide gated cation channel TAX-2/TAX-4 is an example of a protein that is necessary for many sensory processes. TAX-2/TAX-4 is widely expressed in sensory neurons. Consistent with this, *tax-2* and *tax-4* mutant animals are defective in soluble and odorant chemotaxis as well as thermotaxis [14], [15]. Guanylyl cyclase *daf-11* mutants have phenotypes similar to *tax-2* and *tax-4* mutants [16], [17]. This suggests that DAF-11 activity generates the cGMP which gates TAX-2/TAX-4 channels. In our assays, *tax-2* and *daf-11* null mutants were impaired for water soluble and odorant chemotaxis to NH_4_Ac (Fig. 2). DAF-11 is likely to function as a heterodimer with another guanylyl cyclase, ODR-1 [18]. ODR-1 is expressed in non-exposed neurons (AWC and AWB) and exposed neurons (ASI, ASJ and ASK). *odr-1* mutants are defective in AWC and AWB-mediated olfaction but chemotaxis to soluble compounds detected by non-exposed neurons has not been well-studied [19]. In NH_4_Ac chemotaxis assays *odr-1* mutants have significant defects only in odorant assays (Fig. 2B). The G-alpha subunit ODR-3 is mainly involved in sensing odorants and noxious stimuli whereas NaCl sensation is normal [20]. Consistent with this, *odr-3* mutants showed significantly reduced chemotaxis to NH_4_Ac only in the odorant assay (Fig. 2B). These results show that NH_4_Ac sensation depends on G-protein signaling pathways.

(3) Neuron specification mutants. These mutants lack transcription factors which are necessary for correct cell specification [21]. *che-1* has lost all ASE specific expression [22], [23] and *odr-7* has impaired AWA function and morphology [24]. Neither *che-1* nor *odr-7* null mutants showed defects in either type of chemotaxis assay to NH_4_Ac. Thus, perturbing ASE or AWA in isolation does not disrupt NH_4_Ac sensation (Fig. 2). *ceh-36* is a *otx/otd* homeobox gene, which is broadly expressed during embryonic development but in adults is restricted to AWC and ASE [25], [26]. *ceh-36* animals are defective in AWC mediated olfaction[26] but the role of CEH-36 in ASE is unclear. Specifically, it is not clear whether *ceh-36* mainly affects ASE left/right asymmetry[26] or functional properties of ASE [25]. In our assays, *ceh-36(ks86)* and *ceh-36(ky646)* mutants were the only tested mutants completely defective for both water soluble and odorant chemotaxis to NH_4_Ac (Fig. 2). One interpretation of these results is that only ASE and AWC sense NH_4_Ac. Alternatively, *ceh-36* might function more broadly and NH_4_Ac sensation could be distributed across several sensory neurons. To test whether NH_4_Ac sensation involves other olfactory neurons, we assayed the double mutant *odr-7 odr-1* which should be impaired in AWC, AWB and AWA function through a combination of loss of sensory transduction (AWC+AWB) and neuronal specification (AWA) [2], [24]. The *odr-7 odr-1* double mutant showed defects more severe than *odr-1*, although the effect was confined to the odorant assay (Fig. 2). We also constructed a *che-1; odr-7* double mutant in which ASE and AWA function should be impaired. This strain showed no defect in chemotaxis to NH_4_Ac in either water soluble chemotaxis or odorant assays (Fig. S1A). As a control, we generated a *che-1; odr-1* double mutant to impair AWC and ASE function together. We expected this strain to behave similarly to the *ceh-36* mutant, but surprisingly, the *che-1; odr-1* strain showed no significant defect in chemotaxis to NH_4_Ac in water soluble chemotaxis assays and only a partial defect in odorant assays that was similar to the defect of the *odr-1* single mutant (Fig. S1B). Thus, *ceh-36* impairs AWC and ASE function differently than the *che-1; odr-1* double, or *ceh-36* also acts in cells other than AWC and ASE. These results suggest a model in which NH_4_Ac sensation is distributed across several neurons; identification of the specific cells will require laser cell ablations or cellular imaging techniques.

In summary, mutant analysis suggests that both exposed and non-exposed sensory neurons contribute to wild-type NH_4_Ac chemotaxis. Sensory transduction depends on *tax-2*, *daf-11*, and *odr-1*, although there is still a residual response in these mutant backgrounds (Fig. 2). In both water soluble and odorant assays there is a degree of redundancy; only mutations affecting more than one cell significantly impair soluble chemotaxis.

### Acetate chemotaxis is *tax-2/tax-4* independent

**Figure 3:**
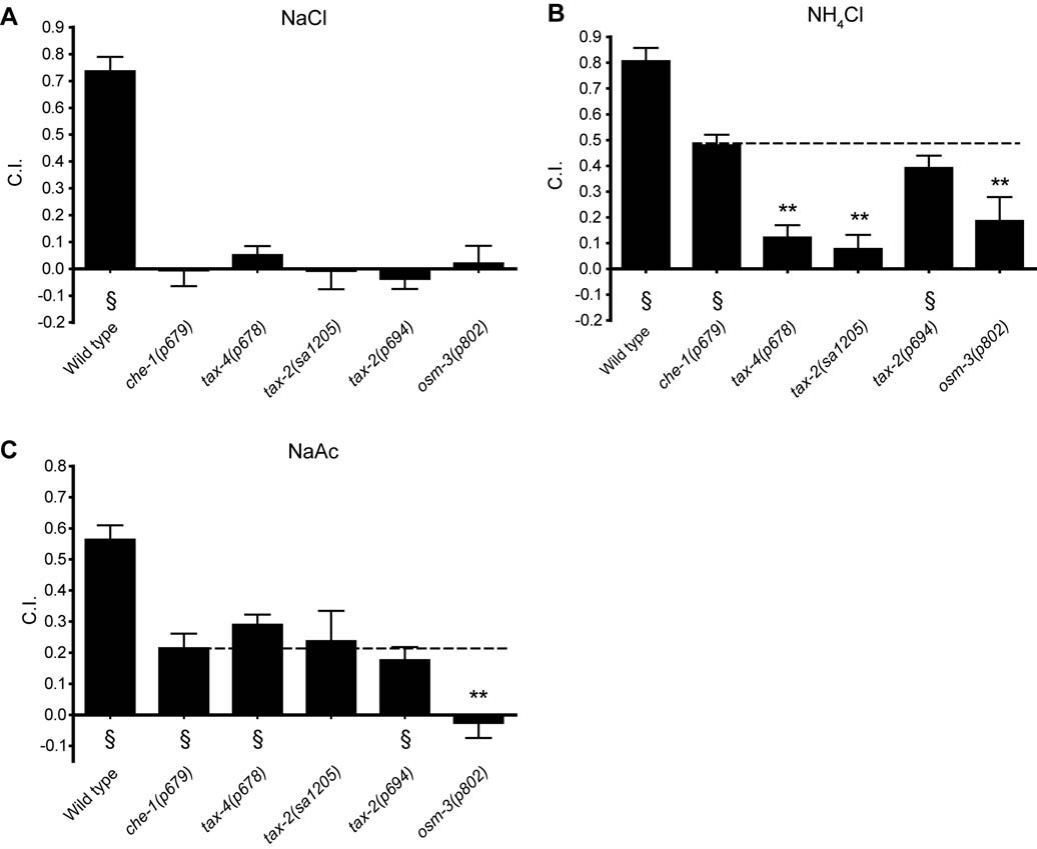
Genetic analysis of ammonium and acetate signal transduction pathways. (A–C) Water soluble chemotaxis assays for NaCl, NH_4_Cl, and NaAc. In each case, radial gradients of attractant were established by diffusion in the agar. Each bar represents the mean of at least 4 independent assays. Statistics: * p<0.05 and ** p<0.01 in a one-way ANOVA and Dunnet's post test comparing all mutants to the *che-1*(p679), which serves as a control in that it has normal chemotaxis to NH_4_Ac but no chemotaxis to NaCl; § p<0.01 in a one sample *t*-test comparing the observed mean to a mean of zero.

To study ammonium and acetate sensation in more detail, we performed water soluble chemotaxis assays with *che-1*, *tax-2*, and *tax-4* in several conditions. Each of these mutants was completely defective in NaCl chemotaxis (Fig. 3A). However, as noted previously, *che-1* mutants had no defect in chemotaxis to NH_4_Ac (Fig. 2A, B). Thus, we interpret *che-1* attraction to NH_4_Cl as chemotaxis to ammonium only, and *che-1* attraction to NaAc as chemotaxis to acetate only. Accordingly, we use the *che-1* strain as our positive control for chemotaxis to these compounds. *tax-4(p678)* and *tax-2(sa1205)* chemotaxis to ammonium was significantly reduced compared to *che-1(p679)* (Fig. 3B). In contrast, acetate chemotaxis was not significantly impaired for either *tax-4* or *tax-2* (Fig. 3C). We furthermore tested two additional alleles of *tax-2(p671* and *p691*) which were both in agreement with *tax-2(sa1205)* (data not shown). Thus, ammonium sensation depends on TAX-2/TAX-4, but acetate is detected by a TAX-2/TAX-4 independent pathway.

We used *tax-2(p694)* as an alternative way to examine which cells are involved in detecting ammonium. In wild-type animals, TAX-2 is expressed in AWC, AFD, ASE, ASG, ASJ, AQR, BAG, ASK, ASI, AWB, and PQR [14]. However, *tax-2(p694)* has a deletion in the promoter region and first exon of *tax-2* that abolishes its expression in only four pairs of neurons: ASE, AQR, AFD, and BAG [14]. *tax-2(p694)* had no defect in chemotaxis to ammonium (Fig. 3B), indicating that these four cells were not necessary for ammonium sensation.

The residual ammonium and acetate chemotaxis ability of *che-1(p679)*, in which ASE neurons are defective, implies that at least one additional sensory neuron is required for chemotaxis to these compounds. To determine whether this additional sensory function resides among the exposed or non-exposed class of neurons, we tested *osm-3(p802)*, in which all exposed cilia are defective, but non-exposed cilia are intact [12]. *osm-3(p802)* was completely defective in sensing NaCl (Fig. 3A) and thus should give ammonium or acetate specific responses in NH_4_Cl and NaAc assays, respectively. We found that *osm-3(p802)* chemotaxis to ammonium and acetate was reduced relative to *che-1(p679)* (Fig. 3B, C). We conclude that some of the residual ammonium and acetate sensory function likely resides among the exposed chemosensory neurons other than ASE. We did not examine further which other exposed neurons might be responsible for the residual responses to ammonium or acetate, though the ADF, ASI, and ASG neurons implicated in NH_4_Cl sensation by laser ablation experiments are possible candidates [1].

### NaAc and NH_4_Cl are not Na^+^ and Cl^−^ specific stimuli

**Figure 4:**
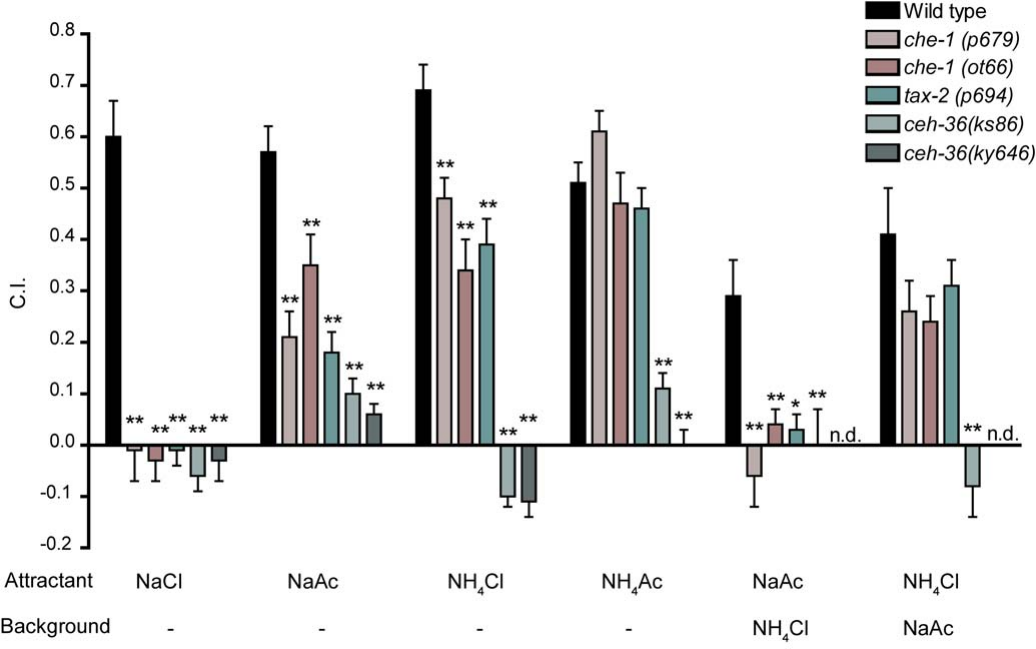
Genetic analysis of the relative ionic contributionsto water soluble chemotaxis assays. Attractants and uniform background compositions are indicated below each group of bars; the no-background conditions are indicated by “-” and n.d. means no data. Each bar represents the mean of at least 8 independent assays. Statistics: * p<0.05 and ** p<0.01 in a one way ANOVA and Dunnet's post test comparing all means to the wild-type (N2) mean.

NaAc and NH_4_Cl have been used as approximations for sodium and chloride specific stimuli in chemotaxis assays 1,27–29 under the assumption that ammonium and acetate are relatively unattractive to worms [3]. However, as noted above, whether or not NH_4_Ac is attractive depends on assay method. We used *che-1*, *tax-2(p694)* and *ceh-36* mutants in water soluble chemotaxis assays to determine the attractive properties of ammonium and acetate ions under our conditions. None of the mutants chemotaxed to NaCl whereas all but *ceh-36* chemotaxed normally to NH_4_Ac and exhibited significant chemotaxis to NaAc and NH_4_Cl (Fig. 4). Because *che-1* and *tax-2(p694)* mutants are completely defective in sensing chloride (see also Fig. 3A) but can still sense ammonium (Fig. 3B), these results indicate that NH_4_Cl, a putative chloride-specific stimulus, is in fact a combination of attraction to both chloride and ammonium under these experimental conditions. Similarly, chemotaxis to NaAc in wild-type animals is a combination of attraction to Na^+^ and acetate.

Although ammonium and acetate were attractive under our conditions, we did find special conditions under which NaAc acts as a Na^+^-only stimulus. In discrimination assays, in which a NaAc gradient is formed on top of a high uniform background concentration of NH_4_Cl, wild-type animals were able to locate the gradient peak (Fig. 4, NaAc/NH4Cl). In contrast, the NaCl-chemotaxis defective mutants *che-1(p679)*, *che-1(ot66)*, and *tax-2(p694)* were unable to locate the gradient peak. This difference between wild-type and NaCl-chemotaxis defective mutants indicates that under conditions of high background NH_4_Cl, wild-type worms are orienting to Na^+^ but not acetate.

Under the opposite conditions, in which NH_4_Cl chemotaxis was tested against a high background of NaAc, the NaCl-chemotaxis defective mutants were not different from wild-type. Thus, these conditions do not provide a Cl^−^-only stimulus.

We also tested two alleles of *ceh-36* (*ks86* and *ky646*) to compare with mutants that have left/right bilateral asymmetries in ASE tested under similar or identical conditions [9], [28]. *ceh-36(ks86)* and *ceh-36(ky646)* were completely impaired in chemotaxis to all attractants, including NaCl (Figure 4). This is in contrast to ASEL/ASER mutants [9], [28], which show relatively subtle defects in chemotaxis to NaAc and NH_4_Cl, but no defects to NaCl. These results suggest that although ASE neurons express most cell specific markers in the *ceh-36* background [22], [25], [26], normal ASE function is abolished.

## Discussion

### Ammonium-acetate is an attractive odorant

*C. elegans* chemosensation has been successfully studied with behavioral assays that treat volatile and water soluble chemotaxis as separate senses, analogous to smell and taste. This approach has been successful, partly because distinct groups of sensory neurons mainly mediate responses only to odorants or to water soluble compounds. In several water soluble chemotaxis assays ammonium and acetate have been used either as neutral counter-ions or as attractants [1], [7]–[9], [28], [29]. Here we show that ammonium and acetate are attractive in both water soluble and odorant chemotaxis assays. Over a range of concentrations, NH_4_Ac spotted on a plate or on the lid above the plate was attractive to *C. elegans*. Furthermore, NH_3_ and acetic acid alone were attractive odorants, leading to accumulation of animals near the source of either. It should be pointed out that NH_4_Ac was used at high concentrations in the odorant assays, probably above the normal range encountered naturally. It is also unlikely that the odorant properties of NH_4_Ac affect the response of *C. elegans* in the standard chemotaxis assay developed by Bargmann and colleagues [1] in which a small point source of attractant is applied the day prior to chemotaxis assays; the local concentration during the chemotaxis assay would not be expected to be high enough to elicit odorant attraction, though it might affect response as a soluble attractant as we have shown. In contrast, the “quadrant assay” developed by Plasterk and colleagues is very different–half of the plate contains attractant at uniform high concentration [7]. Under these experimental conditions it is likely that odorant responses can contribute to NH_4_Ac attraction.

Ammonium and acetate can also be detected as water soluble compounds absorbed into the agar; animals were attracted to the peak of a shallow gradient of water soluble NH_4_Ac where no focal odorant source would be expected because NH_4_Ac has diffused into the agar over a wide area. Furthermore, we used *che-1* animals to test the assumption that NaAc and NH_4_Cl are equivalent to Na^+^ and Cl^−^ specific stimuli. It is clear that this is not a valid assumption under these experimental conditions. On the contrary, we have shown that a significant part of chemotaxis to NaAc and NH_4_Cl is to acetate and ammonium ions (Figure 4).

Chemotaxis to NH_4_Ac appears to conflict with previous findings from our laboratory [28]. The fact that we now find NH_4_Ac to be attractive whereas Pierce-Shimomura *et al*. [28] did not was unexpected because the peak concentration and the spatial extent of the NH_4_Ac gradients were almost identical in the two studies. However, there were three significant differences between the studies. First, in the new assays, worms were immobilized at the gradient peak whereas in our earlier study worms were free to leave the peak, and frequently did so (J. Pierce-Shimomura, personal communication), possibly because of sensory adaptation. Second, we counted the number of worms reaching the peak, whereas Pierce-Shimomura et al. recorded dwell time at the peak. Because dwell time would be reduced by worms leaving the peak, the Pierce-Shimomura assay was probably less sensitive than the present assay. Finally, we performed the assays for 60 minutes on animals started 30 mm from the peak of attractant whereas Pierce-Shimomura et al. assayed single animals for 20 minutes placed 11 mm from the peak.

One question is whether the NH_4_Ac water soluble and odorant assays measure qualitatively different behaviors or are merely quantitatively different measures of the same behavior. Our assays (see Figure 2) are consistent with either possibility. The odorant assay may simply be a more sensitive assay that can reveal the weaker defects of such mutants as *odr-1*, *odr-3,* and *odr-7 odr-1* that were not detectable in water soluble assays. Because we found no mutants that were normal in odorant assays and specifically defective in water soluble assays, we cannot conclude that the two assays measure qualitatively different senses. Whether NH_4_Ac is dissolved in the agar or presented on the lid, there will be an equilibrium between the compound in solution and in the air, and the same cells that “taste” may also “smell” NH_4_Ac, albeit perhaps at different thresholds. The volatility of a compound depends on such factors as its vapor pressure (which in turn is dependent on environmental atmospheric pressure, humidity and temperature) as well as its counter-ion (e.g. the acetate of NH_4_Ac will have greater volatility than the acetate of NaAc because ammonia is volatile and sodium is not). Because worms on an agar plate are surrounded by an aqueous film, a compound will be presented to the amphid neurons in the same milieu regardless of whether it was originally applied as a water soluble or olfactory stimulus. Thus, the distinction between taste and smell in the context of our assays may be largely semantic. We conclude that *C. elegans* is attracted to NH_4_Ac through a combination of volatile and water soluble cues.

### Several cells and distinct pathways detect ammonium and acetate

Our results suggest that NH_4_Ac sensation is distributed over several neurons. Interestingly, impaired ASE and AWC specification in the *ceh-36* mutant completely disrupts NH_4_Ac chemotaxis. Odorant specific mutations *odr-3* and *odr-7 odr-1* which perturb both AWA and AWC disrupt chemotaxis to NH_4_Ac when it is placed on the lid. These mutations do not disrupt water soluble chemotaxis to NH_4_Ac, which probably reflects the bias of the assays—in the lid assay the olfactory sensory component is more heavily weighted. This is supported by additional data: a high uniform background of NaCl perturbs water soluble chemotaxis more than chemotaxis to odorants spotted on the lid (Fig. S2).

At the level of sensory neurons, there is enough redundancy that only mutations affecting at least two of the three pairs of neurons ASE, AWC, and AWA disrupt chemotaxis. However, double mutants of *che-1* with *odr-7* or *odr-1* indicate that this “two of three” model is not correct in its simplest form. One caveat to these experiments is that the mutants used may not completely eliminate the function of the cell. For example, *odr-1* mutations eliminate a single signal transduction component in AWC and should not affect other possible *odr-1* independent signal transduction pathways in this cell. The *che-1* and *odr-7* mutations lack proper terminal differentiation of ASE and AWA. However, in both cases, the cell is not eliminated and may still be capable of some sensation, possibly as a result of acquiring certain features of another sensory neuron. There is some evidence that the default olfactory neuron cell fate resembles AWC [30]. Thus, the effects of the *che-1* and *odr-7* mutations may be less severe than complete elimination of the cell. Additionally, experiments with *osm-3* indicate that exposed sensory neurons other than ASE may also be involved in sensing ammonium and acetate. Therefore, single animal experiments using cell specific laser ablations or calcium imaging are needed to fully characterize the cells involved in detecting NH_4_Ac.

Genetic analysis showed that NH_4_Ac and NaCl chemotaxis are separable processes. *che-1* mutants are unable to chemotax to NaCl yet show wild-type chemotaxis to NH_4_Ac. Effectively, this made it possible for us to use Na^+^ and Cl^−^ as neutral counter-ions for acetate and ammonium ions, respectively. Interestingly, ammonium sensation depends on the TAX-2/TAX-4 channel, but acetate sensation does not. TAX-2/TAX-4 independent sensory pathways are well described in the AWA neurons, where OSM-9 (a TRP-like channel) is necessary for sensory transduction [31]. However, we think it unlikely that attraction to acetate is exclusively mediated by AWA since *odr-7* animals show wild-type chemotaxis to NH_4_Ac. Also, *osm-9* mutants are not defective in chemotaxis to acetic acid (data not shown).

We also assayed the *tax-2(p694)* mutant which has lost TAX-2 expression in ASE, AQR, AFD, and BAG [14]. *tax-2(p694)* has normal AWC function but impaired ASE and AFD function [14]. *tax-2(p694)* chemotaxis to ammonium is not reduced and thus ASE is not necessary for ammonium sensation. Since *ceh-36* chemotaxis is completely impaired, we think it likely that AWC is involved in detecting ammonium in water soluble chemotaxis assays. Cyclic nucleotide dependent signaling in AWC depends on at least two G-alpha subunits, ODR-3 and GPA-3, and two downstream guanylyl cyclases, ODR-1 and DAF-11 [32]. *odr-3*, *odr-1*, *daf-11,* and *tax-2* all show similarly reduced chemotaxis to NH_4_Ac in the odorant assay. Our results are consistent with the interpretation that this reduction is due to a selective loss of ammonium sensation.

Water soluble NH_4_Cl chemosensation has mainly been ascribed to ASE based on the careful analysis of chemotaxis after ablation of all the exposed ciliated neurons, alone or in combination [1]. Comparison of ablated animals with the cilium structure mutant *che-2* (which lacks exposed and non-exposed ciliated neurons), shows that there is a residual response to NH_4_Cl after ablation of ASE (and any of the other exposed neurons). This observation is therefore consistent with a possible role for AWC in ammonium sensation.

### Sensory pathways and taste adaptation

Worms pre-exposed to a compound often have reduced chemotaxis to the same compound, a process termed adaptation. Jansen *et al.* [8] showed that for water soluble compounds this process is partly salt specific. However, not all salts produce adaptation, and cross-adaptation is limited to certain salts. For example, pre-exposure to NH_4_Ac does not cause adaptation to NH_4_Ac or cross-adaptation to NaCl. Adaptation to NaCl appears to be a complex process involving multiple cells and molecular pathways [33]. Our results suggest that multiple sensory cells (ASE, AWC, AWA, and possibly others), at least two separate pathways (TAX-2/TAX-4 dependent and independent), and both the worm equivalents of taste and smell are involved in detecting ammonium and acetate. This could explain the rather complex pattern of partial adaptation and cross-adaptation. Specifically, the lack of cross-adaptation between NH_4_Ac and NaCl is not surprising given that the two salts are sensed by separate pathways.

It is clear that some chemosensory specificity is concentration dependent. For example, *odr-10* mutants which lack the putative diacetyl receptor are unable to locate the peak of low concentrations of diacetyl, but show wild-type chemotaxis to higher concentrations [34]. Similar mechanisms may be important for water soluble chemotaxis. Thus, for chemotaxis to NaCl in gradients with high peak concentrations (25–800 mM), ablation of ASE does not completely eliminate chemotaxis. Under these experimental conditions, ADF, ASG, and ASI are important for NaCl chemotaxis [1]. However, in a modified chemotaxis assay with shallower gradients (peak approx. 10 mM), ablation of ASE eliminates NaCl chemotaxis ([35] and J. Pierce-Shimomura, personal communication). Also, the data presented in this study in a similar shallow gradient showed that two alleles of *che-1* were completely defective in chemotaxis to NaCl, even though *che-1* appears not to be expressed in ADF, ASG or ASI [23]. It is interesting that the *ceh-36* loss of function alleles are not only completely impaired for NH_4_Ac chemotaxis but also for NaCl chemotaxis. *ceh-36* loss of function mutations affect the expression of ASE specific markers only weakly [25], [26] and have been proposed by Lanjuin *et al.*, [26] to mainly affect bilateral asymmetry in the ASE neurons. Our results favor the interpretation by Koga & Ohshima that CEH-36 is necessary for ASE function.

## Materials and Methods

### Strains and genetics

All strains were derived from the wild-type N2 strain and grown under standard conditions at room temperature on nematode growth medium seeded with the *Escherichia coli* strain OP50 [36]. The following mutant strains were used: *ceh-36(ks86) X, ceh-36(ky646) X, che-1(ot66) I, che-1(p679) I, che-2(e1033) X, che-3(e1124) I, daf-11(sa195) V, odr-1(n1936) X, odr-3(n2150) V, odr-7(ky4) X, osm-3(mn391) IV, osm-3(p802) IV, tax-2(p671) II, tax-2(p691) II, tax-2(p694) II, tax-2(sa1205) II, tax-4(p678) III* and the double mutants *kyIs140 I ceh-36(ky646) X, odr-7(ky4) odr-1(n1936) X.* Putative null alleles: *ceh-36(ky646), che-1(p679)*, *che-2(e1033), che-3(e1124), daf-11(sa195), odr-7(ky4), osm-3(p802), tax-2(sa1205)* and *tax-4(p678)* have nonsense mutations in the genes and are putative null alleles [7], [10], [15], [16], [23], [24], [26], [37], J. Kemner, personal communication. *che-1(ot66)* has a deletion of part of the promoter and beginning of gene and is a putative null allele [22]. Loss of function alleles: *odr-3(n2150)* and *osm-3(mn391)* have late nonsense mutations [20], [37], *ceh-36(ks86)* has a missense mutation [25] and *odr-1(n1936)* has a splice donor mutation [19]. *tax-2(p694)* has a deletion in the promoter region and first exon of *tax-2* that abolishes its expression in only four pairs of neurons: ASE, AQR, AFD, and BAG [14].

### Chemotaxis assays

The chemotaxis assay was based on assays developed by Bargmann and Horvitz [1] and Pierce-Shimomura *et al.* [28]. Assays were performed on 10 cm plates containing 20 g/L agar, 5 mM potassium phosphate (pH =  6.0), 1 mM CaCl_2_, and 1 mM MgSO_4_ (“standard plates”). Assay plates for discrimination assays additionally contained 50 mM NaAc, pH = 6.0 or 100 mM NH_4_Cl, pH = 6.0. Different background concentrations of NH_4_Cl and NaAc were used because animals showed poor chemotaxis to NH_4_Cl in 100 mM NaAc [9]. We also tested the effect of assay plate composition in accordance with other published chemotaxis assays: “Jansen” (20 g/L agar, 5 mM potassium-phosphate (pH = 6.6), 1 mM CaCl_2_, 1 mM MgSO_4_ [8]), “Ward” (15 g/L agarose, 10 mM HEPES (pH = 7.2), 0.25% Tween 20 [3]) and “Pierce” (17 g/L agar, 2 mM NH_4_Cl, 1 mM CaCl_2_, 1 mM MgSO_4_, 25 mM potassium-phosphate (pH = 6.5) [28]). Please see figure S3.

Water soluble chemotaxis assays: Radial gradients were formed by placing 10 µL of 2.5 M attractant or ddH_2_O (control) at diametrically opposed locations on the plate (see Fig. 1A). The attractant was allowed to diffuse for 14–16 hours at room temperature. To increase the steepness of the gradient, 4 to 4.5 hours prior to the chemotaxis assay, an additional 4 µL of attractant or ddH_2_O was added to the attractant and control spots, respectively. The peak of the gradient was estimated to be on the order of 10 mM with a fall-off to less than 1 mM at 20 mm from the peak, based on a diffusion model assuming no borders [28]. Attractants NaCl, NH_4_Ac, NH_4_Cl, and NaAc (Sigma, MO, USA) were dissolved in ddH_2_O to a concentration of 2.5 M and adjusted to pH = 6.0 with either ammonium-hydroxide or acetic acid.

Odorant chemotaxis assays: Attractant solution was placed on the lid above the “attractant spot” and ddH_2_O placed above the “negative control spot” immediately before the assay (see Fig. 1A). Attractants for odorant assays were dissolved in ddH_2_O at a concentration of 7.5 M. NH_4_Acetate used directly on the plate was adjusted to pH = 6.0. For the dose-response curve in Fig. 1B, the odorant was placed directly on the plate immediately prior to placing worms on the plate. For dose-response odorant chemotaxis assays (Fig. 1B and C) odorant concentration was kept constant and different volumes of attractant were placed on the assay plate.

For both types of assay, synchronized unstarved adult animals were rinsed off culture plates with S basal for odorant assays and sterile ddH_2_O for water soluble chemotaxis assays. To remove bacteria and other potential attractants, animals were subsequently washed twice with 10 mL ddH_2_O and pelleted loosely in a table top centrifuge. Animals were transferred using glass Pasteur pipettes. The rinse and wash procedure took ∼15–20 minutes. Before placing animals on assay plates, sodium azide (2.0–2.5 µL, 0. 25 M) was pipetted onto the plate at the attractive spot and the negative control spot to immobilize animals reaching either spot. The azide immobilized animals within a radius of ∼10 mm. Animals were transferred to the center of the plate in a droplet of ∼50 µL ddH_2_O. Excess ddH_2_O was removed with filter paper. Chemotaxis assays were performed at room temperature for 60 minutes and assay plates were subsequently placed in a refrigerator (5°C) to prevent further movement of the animals. Results were quantified by counting worms that reached the attractant spot (zone A), the negative control spot (zone C), or the remainder of the plate (zone B), as shown in Fig. 1A. Animals that were found in the inner circle at the end of the assay period were counted but not included in the count of total number of animals, because most of these animals were injured, dead, or had burrowed in the agar. Chemotaxis index (C.I.) was calculated as (A−C)/(A+B+C). The theoretical range of the index was 1.0 (complete attraction) to −1.0 (complete repulsion). There were usually ∼150 worms per plate; plates with less than 30 worms were not counted. In general, two assays with the same attractant were performed in parallel with the two plates oriented in opposite directions to minimize the influence of extraneous cues.

We did note one qualitative difference between chemotaxis toward NH_4_Ac or acetic acid and the other compounds. Animals were attracted to NH_4_Ac and acetic acid yet never reached the peak of the gradient; instead, animals were paralyzed a small distance away. Also, when stored at 5°C, the animals appeared to decompose faster on plates containing NH_4_Ac or acetic acid than on plates containing the other attractants. The high concentration of acetate was not sufficient in itself to paralyze the animals, because nematodes reached the attractant peak on plates without azide. Thus acetate appears to sensitize worms to the effect of azide.

### Statistics

Means represent data pooled from assays run on at least three different days; error bars are s.e.m.. Methods for specific statistical comparisons are given in the figure legends.

## Supporting Information

Figure S1 **NH_4_Ac odorant chemotaxis of double mutants.** (A) *che-1(p679)*; *odr-7(ky4)* double mutant chemotaxis. (B) *che-1(p679)*; *odr-1(n1936)* double mutant chemotaxis. Only four assays were performed and therefore no statistical analysis has been performed on these experiments.
(0.94 MB TIF)

Figure S2 **Effects of salts in plate on NH_4_Ac chemotaxis.** (A) N2 water soluble chemotaxis to NH_4_Ac with normal chemotaxis plates (background “blank”), 50 mM Na-acetate or 100 mM NH_4_Cl or 100 mM NaCl added to chemotaxis plate. (B) N2 odor-lid chemotaxis to volumes of 7.5 M NH_4_Ac spotted on lid on standard chemotaxis plates (background “blank”) or 100 mM NaCl (background “NaCl”). Statistics: Each data point represents the mean of at least 5 independent assays, error bars represent SEM. Statistics: (C) and (D) One-way ANOVA and Tukey's multiple comparisons test between all pairs of columns.
(1.00 MB TIF)

Figure S3 **Effect of plate composition on NH_4_Ac chemotaxis.** (A) N2 odor chemotaxis to 10 µL 7.5 M NH_4_Ac spotted on plate before assay. Four different types of chemotaxis plates were used (see Materials and Methods) There is no statistical difference between means. (B) N2 odor-lid chemotaxis to 10 µL 7.5 M NH_4_Ac spotted on lid. The effect of plate composition is small, except for “Ward” background, which is statistically different from all other backgrounds. Worms moved very poorly on agarose plates and it is not clear if the low chemotaxis index represents a lack of NH_4_Ac sensation or a movement defect. Statistics: Each data point represents the mean of at least 5 independent assays, error bars represent SEM. Statistics: (C) and (D) One-way ANOVA and Tukey's multiple comparisons test between all pairs of columns.
(1.36 MB TIF)
