# Supplementary figures and images for: Ammonium-Acetate Is Sensed by Gustatory and Olfactory Neurons in Caenorhabditis elegans
Source: PLoS One. 2008 Jun 18;3(6):e2467. doi: 10.1371/journal.pone.0002467 (PMC2413426; doi:10.1371/journal.pone.0002467)

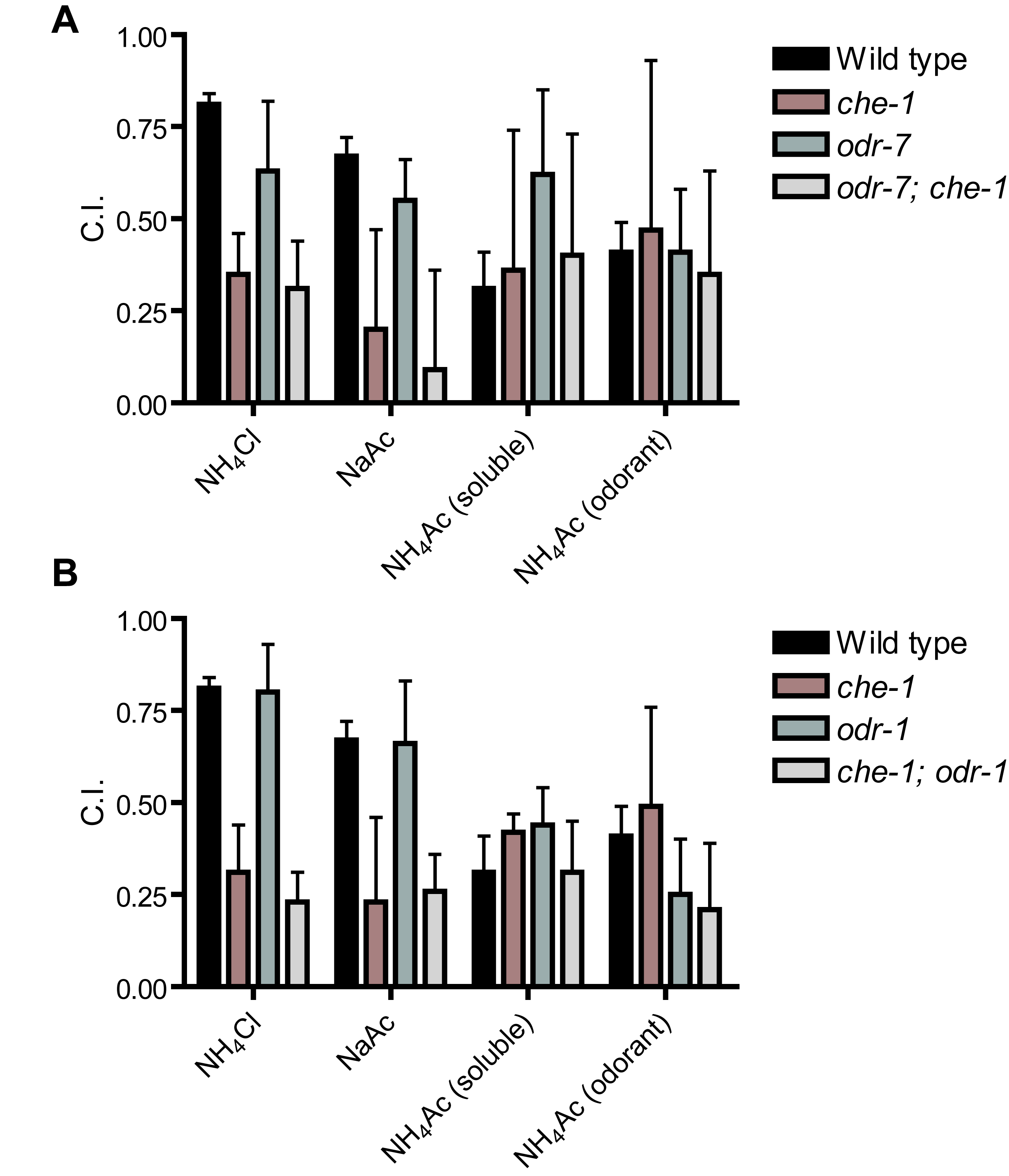

Supplement: Figure S1 — NH4Ac odorant chemotaxis of double mutants. (A) che-1(p679); odr-7(ky4) double mutant chemotaxis. (B) che-1(p679); odr-1(n1936) double mutant chemotaxis. Only four assays were performed and therefore no statistical analysis has been performed on these experiments. (0.94 MB TIF) [file pone.0002467.s001.tif]

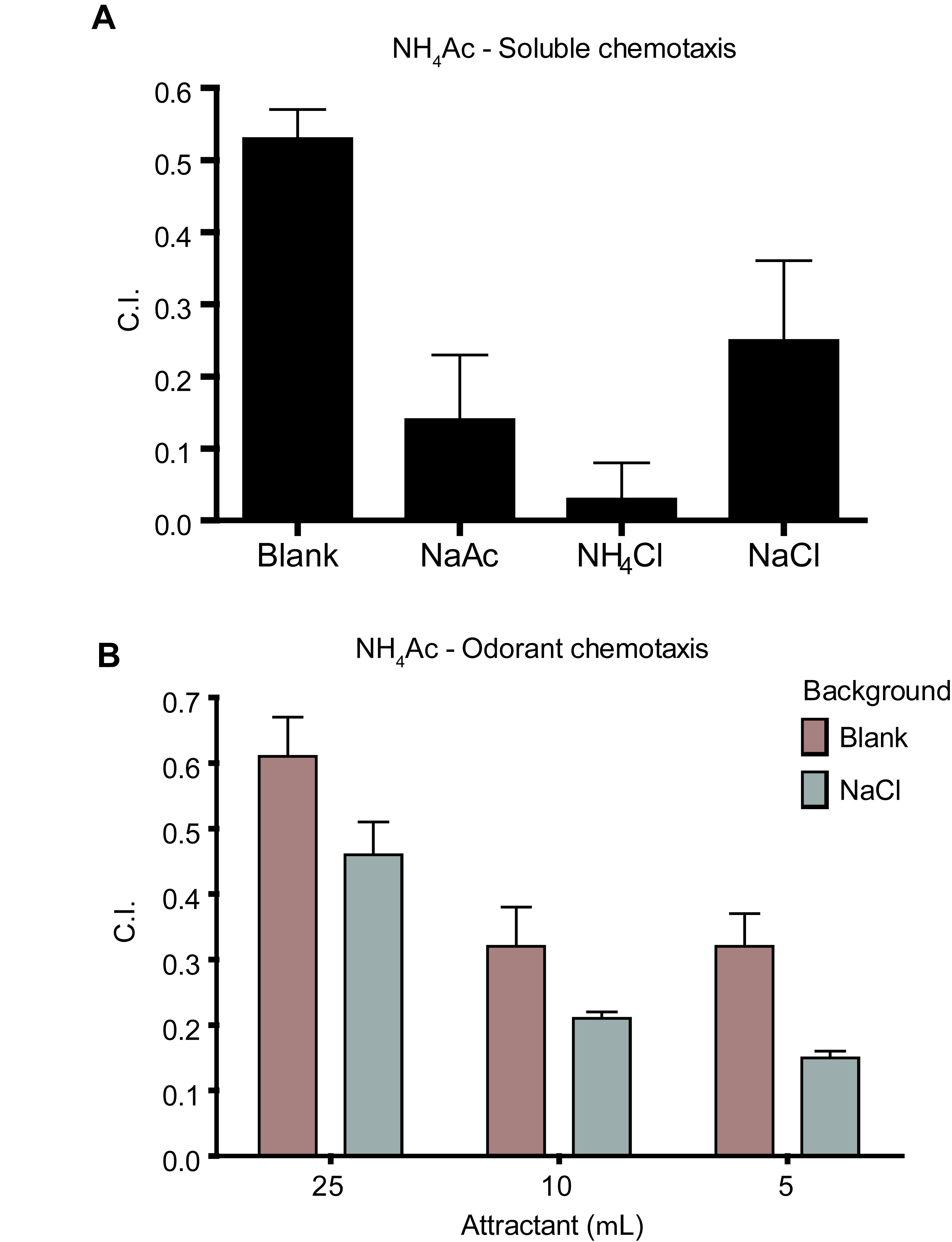

Supplement: Figure S2 — Effects of salts in plate on NH4Ac chemotaxis. (A) N2 water soluble chemotaxis to NH4Ac with normal chemotaxis plates (background “blank”), 50 mM Na-acetate or 100 mM NH4Cl or 100 mM NaCl added to chemotaxis plate. (B) N2 odor-lid chemotaxis to volumes of 7.5 M NH4Ac spotted on lid on standard chemotaxis plates (background “blank”) or 100 mM NaCl (background “NaCl”). Statistics: Each data point represents the mean of at least 5 independent assays, error bars represent SEM. Statistics: (C) and (D) One-way ANOVA and Tukey's multiple comparisons test between all pairs of columns. (1.00 MB TIF) [file pone.0002467.s002.tif]

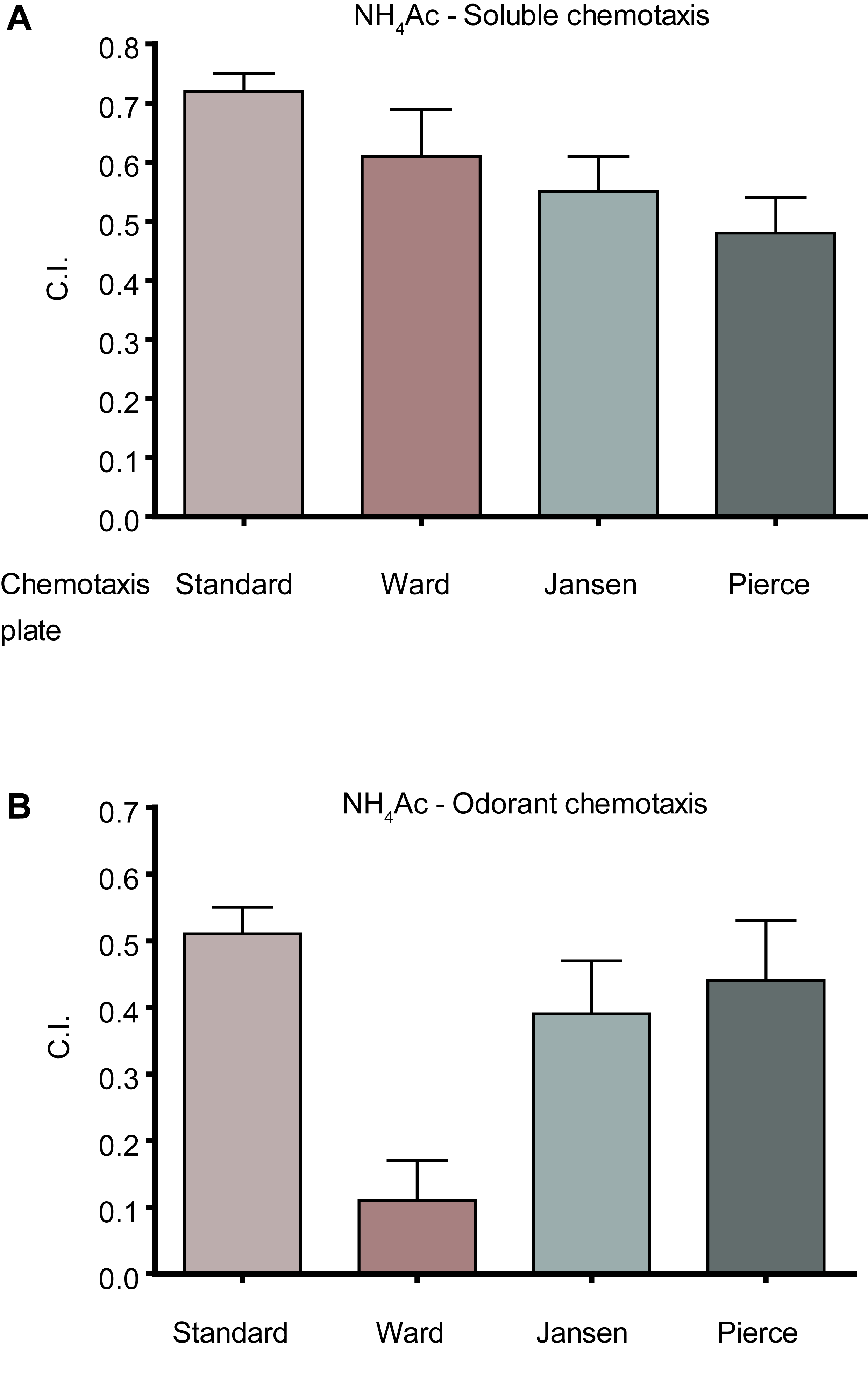

Supplement: Figure S3 — Effect of plate composition on NH4Ac chemotaxis. (A) N2 odor chemotaxis to 10 µL 7.5 M NH4Ac spotted on plate before assay. Four different types of chemotaxis plates were used (see Materials and Methods) There is no statistical difference between means. (B) N2 odor-lid chemotaxis to 10 µL 7.5 M NH4Ac spotted on lid. The effect of plate composition is small, except for “Ward” background, which is statistically different from all other backgrounds. Worms moved very poorly on agarose plates and it is not clear if the low chemotaxis index represents a lack of NH4Ac sensation or a movement defect. Statistics: Each data point represents the mean of at least 5 independent assays, error bars represent SEM. Statistics: (C) and (D) One-way ANOVA and Tukey's multiple comparisons test between all pairs of columns. (1.36 MB TIF) [file pone.0002467.s003.tif]
